# Security Baseline for Substation Automation Systems

**DOI:** 10.3390/s23167125

**Published:** 2023-08-11

**Authors:** Josef Horalek, Vladimir Sobeslav

**Affiliations:** Department of Information Technologies, Faculty of Informatics and Management, 500 03 Hradec Kralove, Czech Republic; josef.horalek@uhk.cz

**Keywords:** security baseline, energy, substation, ISO 27001, smart grid

## Abstract

The use of information technology and the automation of control systems in the energy sector enables a more efficient transmission and distribution of electricity. However, in addition to the many benefits that the deployment of intelligent and largely autonomous systems brings, it also carries risks associated with information and cyber security breaches. Technology systems form a specific and critical communication infrastructure, in which powerful control elements integrating IoT principles and IED devices are present. It also contains intelligent access control systems such as RTU, IDE, HMI, and SCADA systems that provide communication with the data and control center on the outer perimeter. Therefore, the key question is how to comprehensively protect these specialized systems and how to approach security implementation projects in this area. To establish rules, procedures, and techniques to ensure the cyber security of smart grid control systems in the energy sector, it is necessary to understand the security threats and bring appropriate measures to ensure the security of energy distribution. Given the use of a wide range of information and industrial technologies, it is difficult to protect energy distribution systems using standard constraints to protect common IT technologies and business processes. Therefore, as part of a comprehensive approach to cyber security, specifics such as legislative framework, technological constraints, international standards, specialized protocols or company processes, and many others need to be considered. Therefore, the key question is how to comprehensively protect these specialized systems and how to approach security implementation projects in this area. In this article, a basic security concept for control systems of power stations, which are part of the power transmission and distribution system, is presented based on the Smart Grid domain model with emphasis on substation intelligence, according to the Purdue model. The main contribution of the paper is the comprehensive design of mitigation measures divided into mandatory and recommended implementation based on the standards defined within the MITRE ATT&CK matrix specified, concerning the specifications of intelligent distribution substations. The proposed and industry-tested solution is mapped to meet the international security standards ISO 27001 and national legislation reflecting the requirements of NIS2. This ensures that the security requirements will be met when implementing the proposed Security Baseline.

## 1. Introduction

Information security and protection is now a widely resonating topic that is not only being addressed by many research teams and commercial companies around the world but also has significant support from government and international organizations, contributing to a general awareness of security issues in the digital world. The energy sector is not left out of this perspective. Modern power substation automation systems, which are one of the core components of the smart grid [1], are based on the requirement to obtain and use comprehensive data from the Intelligent Electronic Device (IED) devices in use and which are deployed. Therefore, the primary purpose of this requirement stems from the need to ensure maximum unattended operation of substations, the requirement for a reliable and functional system for remote control of individual IEDs, network elements, and their monitoring from the parent High Voltage (HV), Very High Voltage (VHV) network operation control centers and data centers, ensuring their communication with each other. Another important requirement for control systems is to ensure the autonomous operation of the most important elements and protection of the substation power elements to prevent erroneous manipulations that could lead to damage to health or property, and to prevent malfunctioning of protections leading to unnecessary interruption of the power supply to the grid, and disruption of the functionality of the entire system [2]. With the development of information technology, enterprise information systems, IED devices, and the increase in the quantity and quality of data provided, a wide range of possibilities and applications for substation automation systems are opening [3]. These include the use of data from IED devices to increase the efficiency of power grid operations, enabling efficient use of transmission line capacity, rapid identification of fault locations and, last but not least, the possibility of remote switching of protection settings to change the grid operation mode or remote readings from end-of-line smart meters. Another possibility (and requirement) given by the effective use of IED data is the improvement of maintenance and service planning of important primary parts of the whole network and substation system, which is based on the possibility of statistical evaluation of the operation of network and substation elements and their comparison with guaranteed parameters, e.g., the energy counter switched on or off by the circuit breaker with respect to its parameters.

The possibility of comprehensive remote management of the IED system is also a great benefit. IED devices are nowadays connected directly to the local substation network and thus become the core elements forming the smart grid system. This network can be further connected to the WAN network of the entire power utility. Specialists managing IEDs and substation automation systems can carry out IED management, SW maintenance, and fault assessment from any location with access to the utility’s WAN. This approach allows a significant speed-up in identifying the causes of potential faults and further reduces the number of technicians required to manage the substation automation systems of the utility [4]. For large utilities, this can result in significant savings in personnel costs while maintaining or even improving the quality of maintenance and speed of fault clearance.

A key problem addressed by vendors, distribution companies, as well as auditors, and companies involved in improving security within critical infrastructure, is how to implement security policies most concretely and to ensure a higher level of security for substations that communicate over the traditional TCP/IP networks or Internet. There can be a number of legal measures; at the EU level, it is mainly the Directive (EU) 2016/1148 of the European Parliament and Council and the newly coming Directive (EU) 2022/2555 of the European Parliament and Council, better known as NIS2, which is again expanding the range of affected companies and areas. There are also generally accepted standards, such as ISO/IEC 27001, but these do not include direct measures and do not consider the specifics of substation automation. These sets of standards are also referenced and used by ENISA (European Union Agency for Cybersecurity) to address mitigation measures at a general and often non-specific level. At the technological level, it is possible to use the list of the main assets of industrial systems in the so-called ATT&CK knowledge base for Industrial Control Systems (ICS), extended by the level providing communication with the ICS data center. For the above reasons, this paper aims to propose a security baseline that details the issues of cyber security threats and ways of eliminating these threats within the control systems of substations. The output of this paper is a security baseline that can therefore methodically support security measures based on the risk assessment of individual systems and their technological equipment in real projects. By combining individual measures, it is then possible to achieve not only compliance with legislative regulations but most importantly to efficiently secure an appropriate level of critical infrastructure in the power distribution.

From the above, it is clear that the maximum deployment of intelligent and autonomous systems ensuring their operation and the transmission of control and analytical information is now essential to ensure efficient and secure transmission and distribution of electricity. However, with each step of automation and digitalization, there are emerging risks [5] and system vulnerabilities [6] that need to be adequately addressed [7], and appropriate mitigation measures taken to reduce or eliminate them. Addressing cyber security in the “world” of technological systems, where information and communication technologies (ICT) are combined with industrial control systems (ICS), while at the same time being required to meet the technical and technological requirements specified by ISO and IEC standards, is a non-trivial and complex issue [8] that was the focus of many scientific and research teams collaborating within the industry for many years [9], and is still relevant and open.

Based on these challenges and needs, this article is structured as follows. In the introduction, the issue of ensuring the security of substation automation systems is presented, and the problems, challenges, and benefits are analyzed in more detail. The related works section presents current approaches for substation security, including the current trends of machine learning and many others. The following chapter presents the Substation architecture model. The mode itself is based on the respected MITRE ATT&CK for Industrial Control Systems (ICS) knowledge base, which is rather more general and not focused on substation automation systems specifics. Based on the construction of the Substation architecture model, it is possible to decompose individual substation levels, determine key assets and define risks and threats in the given areas. The key output is the Security baseline for power distribution control systems, which, with an emphasis on the ISO 27001 wage standard, recommends specific mitigation measures that suppliers can use in substation automation projects.

### Main Contributions

The main contribution of this paper is the design of the Security Baseline, which was developed based on the security needs of control system suppliers such as ABB, HITACHI, or SIEMENS for substation automation. The selected suppliers participated in the actual design and verification within the framework of the Technology Agency of the Czech Republic project. The Security Baseline is based on the analysis of technical solutions and internationally recognized security standards used for the initial cumulative analysis of the solution to the problem of ensuring cyber security of control systems of electrical substations, their sub-type assets, normative threats, and the relevance of the implementation of the proposed and test Security Baselines. As primary and secondary sources were used, the technological and security standards and their impact on legal regulation in the field of cyber security, based on the legal framework defined by the European Commission and their national implementation in the field of critical systems, means that there are control systems of electric power stations that fail. In addition, the proposed solutions were validated both by the demanding cyber security authority and by the industrial partner, which is one of the suppliers of these systems with a significant share of the national and European markets. Based on the analysis of the techniques and tactics used by attackers on ICS systems, uniform threats were mapped against the underlying security standards. Mitigating measures were proposed to address the generalized threats resulting from non-compliance with uniform security standards, which, as a whole, form a security baseline that is mapped to the legislative and normative requirements so that their implementation would meet these requirements.

## 2. Cyber Security in Substation Automation Systems

Ensuring cyber security, especially at power substations and distribution networks, is a complex matter influenced by the specifics of the industry. Security standards must be met, but they also need to be implemented in such a way that does not affect compliance with the operating conditions defined by operational standards and regulations. In particular, the international communication standard IEC 60870 defines communication protocols designed for remote control systems and SCADA systems. The IEC standard helps to guarantee the interoperability of various systems from different suppliers at the level of service provision and interoperability. Another important standard is the IEC 61850 standard, which defines the rules of communication between devices in substations, the requirements for substations, and their equipment in terms of communication. More than fifty different communication protocols are used for communication at substations. The IEC 61850 is generally a set of standards that represents a unified and standardized method of creating a communication network that is independent of suppliers and, at the same time, represents the process of integration of individual substation equipment. The IED devices, connected via a data network, then allow monitoring of the substation’s devices, implementing automation measurement and control functions. All considered and implemented safety measures must not limit and jeopardize the fulfillment of technical parameters of the mentioned standards, and thus disrupt the transmission of electricity itself, and it is, therefore, advisable to have a set of proven safety measures that will not impair compliance with the above technical standards.

Cyber security in substation automation should respect international standards with an emphasis on industrial technologies. In the field of information security, the most widely used are a series of standards related to the Information Security Management System (hereinafter referred to as ISMS). These ISMS standards are composed of interrelated standards that contain a number of structural components. They can be divided into:

- Standards containing an overview and terminology—ISO/IEC 27000.
- Standards specifying requirements—ISO/IEC 27001, ISO/IEC 27006, ISO/IEC 27009.
- Standards describing general guidelines—ISO/IEC 27002, ISO/IEC 27003, ISO/IEC 27004, ISO/IEC 27005, ISO/IEC 27007, ISO/IEC 27014.
- Standards describing industry-specific guidelines—ISO/IEC 27011, ISO/IEC 27019, ISO/IEC 27799.

The complexity of ensuring cyber security within the smart grid and systems associated with the extensive use of communication infrastructure is discussed by the team led by T. Kraus of Cyber Analysis & Defense, Fraunhofer FKIE in the article “Cybersecurity in Power Grids” [10]. However, this creates more scope for potential cyberattacks on this infrastructure and its services. The authors focus on the analysis of the communication infrastructure of power grids and derive from it the problems of power grids with respect to cyber security. They identify a broad set of attack vectors and attack scenarios that threaten the security of power networks for which they propose to employ a defensively in-depth strategy that includes measures for device and application security, network security, physical security, and ultimately policies, procedures, and information sharing. The common basis for these analyses is the implementation of risk analysis as indicated by V. Mokhor from Pukhov Institute for Modelling in Energy Engineering [11,12], respectively, in the article “Intelligent System for Risk Identification of Cybersecurity Violations in Energy Facility”. Both articles are connected by the detailed concept of complex cyber security risks of information systems of critical infrastructure objects, in the form of a vector risk model, including similarly developed options for calculating the overall risk, based on the structural decision of an autonomous computer system for calculating the cyber security risk of such systems Using the published approaches, it is then possible to assess the risks as a whole, including the consideration of the human subjective factor in risk assessment, which is extremely important for critical infrastructure, especially in the energy sector. The results can then effectively influence the entire system of cyber security risk assessment of information systems of critical infrastructure objects in the construction and implementation of information security management systems, integrated information protection systems in automated systems in the development of threat models, security policy, and protection plan. In the area of cyber security assurance, it is then necessary to take into account the issue of zero trust (ZT) systems, which sometimes need to be incorporated into the smart grid network architecture due to emerging computing technologies, e.g., cloud or edge computing. Incorporating these components into the smart grid contributes to creating more complex, distributed, and dynamic operating environments. To meet the challenges posed by these changes, new enterprise security architectures are emerging, for example, based on the concept of Zero Trust (ZT) as defined by the team around S. Xiao in their paper [13] “SoK: Context and Risk Aware Access Control for Zero Trust Systems”. ZT systems then consider internal and external networks as untrusted, and both are subject to the same security controls and management to prevent data breaches and limit internal lateral movement. In that paper, the authors then conduct a systematic examination of ZT, context awareness, and risk-based access control to explore the critical elements of each and identify areas of overlap and synergy to improve the operation and deployment of ZT systems.

### 2.1. Technological Constraints

The issue of smart grid security, conceived as a system composed of distributed and heterogeneous components, whose task is to intelligently secure the electricity supply, is also addressed by Zakaria El Mrabet of the University of North Dakota, in his article “Cyber-security in smart grid: Survey and challenges” [14]. Here he points out that these systems suffer from many security weaknesses, which he analyses in detail regarding the likelihood of an attack and its impact, and focuses on the characteristics of attacks in smart grids, estimating their impact and proposing countermeasures. The classification of cyberattacks based on four steps, namely reconnaissance, scanning, exploit, and maintaining access, can be considered a significant contribution. These basic steps are generally followed by attackers to compromise any system. Finally, the authors propose a cyber security strategy based on the phases of attacks, i.e., pre-attack defense, attack defense, and post-attack defense. For example, in the first step, several techniques were described for network security, data security, and device security. In the second step, the techniques used for attack detection and mitigation were presented. In the last step, a forensic technique was introduced to identify the subject involved in the attack. Following the authors’ conclusions, it can be concluded that this strategic approach can help to address potential component vulnerabilities, enhance the security of network communications, and protect customer privacy. These conclusions are then confirmed by D. B. Rawat of Howard University in his paper, “Cyber security for smart grid systems” [15]. It outlines the current state of research and future perspectives, allowing for a more thorough understanding of smart grid security and research trends in this area.

There is no doubt that the smart grid is one of the most important applications of the Internet of Things (IoT), as these networks use a wide range of OT elements, among which we can include disconnectors, phasor measurement units, protective relays, RTUs, etc. With the development of information and communication technologies and their applications in power systems, it is necessary to continuously increase and improve the cyber physical protection of smart grid systems. IoT-based systems are becoming a critical infrastructure with a relatively complex and heterogeneous architecture. In this area, the summary study by Z. A. Khan, “Recent Advancements in Intrusion Detection Systems for the Internet of Things” [16], provides an overview of intrusion detection systems (IDS) suitable for IoT networks. 

**Figure 1:**
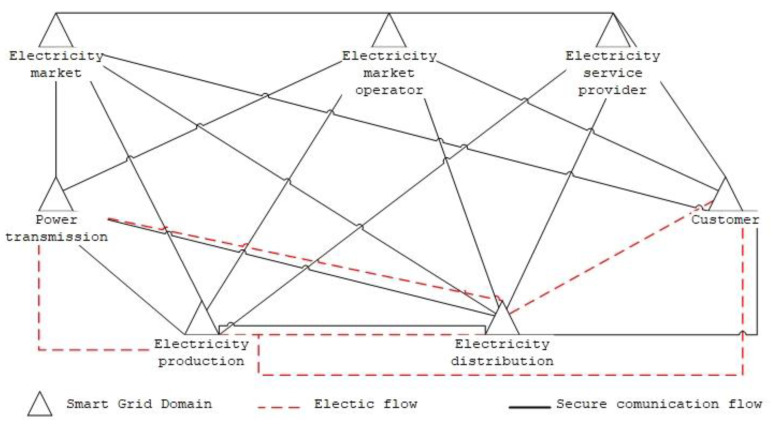
Smart grid conceptual model.

Communications systems are an integral part of the smart grid and, if the confidentiality, integrity, or availability of communications is compromised, they can lead to significant national security impacts, disruption of public order, loss of life, or widespread economic damage. These large-scale systems are vulnerable to cyberattacks. Implementing security approaches is therefore important to improve the overall solution and resilience to cyberattacks. This hypothesis is shared by M. Z. Gunduz from Bingol University and in his paper “Cyber-security on smart grid: Threats and potential solutions” [17], which presents a detailed analysis of threats and potential solutions in IoT-based smart grid environments. We focus on the types of cyberattacks and provide a detailed comprehensive overview of the state of smart grid cyber security. Our work uses the NIST conceptual model of the smart grid, composed of seven functional domains, and outlines linkages at the power transmission and data flow levels. The general conceptual Smart Grid domain model, which is shown in Figure 1, is the commonly used Smart Grid soma model as reported by NIST in [18] and is also used by Lopér et al. in their work, “Multi-Faceted Assessment of a Wireless Communications Infrastructure for the Green Neighborhoods of the Smart Grid” [19], in addition to [17].

The whole concept of Trof smart grid security is based on information security measures using the CIA triad [20]. These key security principles must definitely be met in smart grid systems. It presents the classification of cyberattacks on smart grids according to the CIA triad and the classification of attacks according to the TCP/IP network model. The result is then a comprehensive view of typical attacks in a smart grid network environment that decomposes the target of the attack at each network layer according to the CIA triad, as shown in Table 1.

Smart grid security issues include data collection and control of devices such as PLCs, smart meters, IEDs, RTUs and PMUs. There are also network security issues, including firewalls, attack scenarios, countermeasures, encryption, intrusion analysis, forensics, and routers. Going in the direction of classifying cyberattacks to account for important information security factors provides a clear and useful way to provide practical solutions for current and future attacks on the smart grid. This way of addressing cyber security also allows to view the smart grid from an information security perspective, as defined by the Information Security Management System (ISMS) and according to the ISO 27000 standard, and it is necessary to classify the hitherto partially atomized view of vulnerabilities, threats and impacts from a risk perspective, taking into account the specific focus and solution of the smart grid and then appropriately implementing a set of security measures in order to protect the data and its own, as according to the CIA triad. 

### 2.2. Legal Aspects

The issue of smart grid security is not only the domain of the US and the EU, as evidenced by Ejaz Ul Haq [21]. Current trends in the field of cyber security in the energy sector are significantly influenced by security strategies based on binding regulatory requirements, mainly the directive (EU) 2016/1148 [22]. This directive is concerning measures for a high-common level of security of network and information systems across the Union. The proposal, known as NIS2, and its implementation into national legislation of EU member states, is one of the most discussed topics in the scientific and professional community. Already in the framework of the preparation of this directive, the book, Cybersecurity in the Electricity Sector [23] by R. Leszczyna, in the chapter “The Current State of Cybersecurity in the Electricity Sector”, the evaluation studies published, not only by NIST, but also by The European Union Agency for Cybersecurity (ENISA) are presented. This importance is significantly reinforced in NIS2 with an emphasis on explaining the vulnerabilities of the evolving electricity infrastructure and describing the new threats and risks [24]. Cyber security challenges related to the transformation and corresponding initiatives are described. Directions for future cyber defense efforts in the evolving electric power sector are suggested. Then, in the “Cybersecurity Assessment” chapter, threats are also assessed in light of the rules just outlined in the NIS2.

An erudite description of policies and joint initiatives in the field of cyber security in the European Union, including insights on the need to reconcile ongoing technological advances with regulatory efforts, is provided by J. L. Hernandez-Ramos of the European Commission in his article “Toward a Data-Driven Society: A Technological Perspective on the Development of Cybersecurity and Data-Protection Policies” [25]. A very detailed analysis, not only in the field of regulations governing cyber security in the energy sector, considering interdependencies between sectors and cross-border links within the EU, is provided in the article “Legal Aspects of Cybersecurity in the Energy Sector-Current State and Latest Proposals of Legislative Changes by the EU” [26] by M. Krzykowski from the Faculty of Law and Administration. He points to the problem that the lack of harmonized regulations in this area may not only lead to restrictions in the physical supply of electricity but, in an extreme case, may also make it impossible to achieve the objectives of EU energy policy, in particular, integration within the single energy market and ensuring cyber security.

### 2.3. International Standards

Significant additions and amendments to NIS2 are based on the binding legislative framework of international standards, issued by the International Organization for Standardization (ISO), referred to as ISO standards. It is an organization coordinating standardization technical activities on an international scale aimed at facilitating cooperation in the fields of science, technology, and the economy. Technical committees are responsible for the development of international standards for individual areas of competence. ISO members then vote on the proposals of individual standards. International standards are then used to develop European standards marked “EN”. The standards are then adopted by individual states and after translation into the national language, the standard receives the designation “ČSN”. The national representative of ISO for the Czech Republic is the Czech Standards Institute.

**Figure 2:**
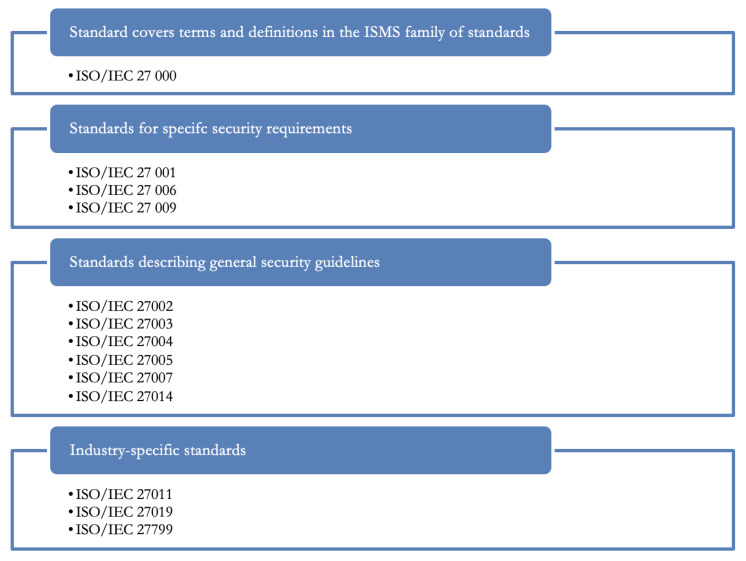
Categorization of international standards in substation automation.

The basic standards in the field of information security include a number of standards related to the Information Security Management System (hereinafter referred to as ISMS). As stated (ÚNMZ, 2020), ISMS standards are composed of interrelated standards that contain a number of structural components. Figure 2 provides a general overview of the links between ISMS standards.

When dealing with comprehensive and effective cyber security of the smart grid, with regard to the aforementioned links of the general ISMS framework, it is imperative to pay attention to the evaluation and systematic training and improvement of the set security system. P. D. Curtis, in his article, “Evaluating and improving cybersecurity capabilities of the energy critical infrastructure” [27], presents the Cybersecurity Capability Maturity Model (C2M2) and two versions of the model adapted for the energy sector—Electricity Subsector Cybersecurity Capability Maturity Model (ES-C2M2) and Oil and Natural Gas Cybersecurity Capability Maturity Model (ONG-C2M2). These are proven tools that enable owners and operators of critical infrastructure elements in the electricity sector to assess their cyber security capabilities and inform their prioritization of actions and investments to improve cyber security. These models and related tools are intended to support the ongoing development and measurement of cyber security capabilities within the electricity and oil and gas subsectors. The models can be used to enhance cyber security capabilities in the sub-sector to effectively and consistently assess and benchmark cyber security capabilities, and share knowledge and best practices to improve cyber security capabilities, and to prioritize actions and investments to improve cyber security. However, these evaluations are only effective when they consider current developments in cyber security and data protection technologies and developments and the dissemination of security awareness. And this is the area that is addressed in detail by [28] in their article “Proliferation of Cyber Situational Awareness: Today’s Truly Pervasive Drive of Cybersecurity”, where they state that the issue of situational awareness (SA) requires an understanding of current activities, the ability to predict what will happen next, and strategies to assess the threat or impact of current Internet activities and forecasts. These SA practices are universal, domain-independent, and can be used to detect cyber breaches. This paper introduces cyber situational awareness (CSA), its origin, concept, objective, and characteristics based on a gap analysis of functions and development requirements. Approaches to CSA assessment were divided by the authors into three methods: mathematical model, knowledge-based reasoning, and pattern recognition. The paper further discusses CSA from three perspectives: model, knowledge representation, and evaluation methods. Future directions for the development of CSA are outlined with an emphasis on gaining information about adversary activities that can be used to improve an organization’s SA operations.

### 2.4. New Trends in Substation Automation Security

Substation automation, as a part of critical infrastructure, helps to ensure stable power management and delivery. Modern cyber security threats and challenges are affecting the substation automation systems, Smart Grids, or any other industrial solutions. Smart substations and modern systems include the implementation of Artificial Intelligence and Machine Learning. The selected and important approaches in this area are presented below.

The integration of demand side management (DSM) and Smart Grid can facilitate the transition of residents to smart homes and sustainable cities by reducing carbon emissions. In [29], Sarker E. et al. provides an overview of recent works related to the application of DSM in SG by discussing the techniques and algorithms and their associated challenges for effective implementation. The paper also critically discusses the operational mode of DSM, the generation, storage, and energy consumption profile, and finally, the benefits obtained by the implementation of DSM. As stated by Lyulyov O. et al. in their paper [30], a comprehensive assessment of Smart Grids is essential for their development. In this paper, the authors analyze the most well-known approaches to the comprehensive assessment of Smart Grids, according to the completeness of their coverage of the most critical components. In the paper, the authors identify the key areas for Smart Grid assessment according to the most cited and relevant research and regulatory documents of the European Union, including the area of security. A comprehensive overview of the use of artificial intelligence techniques in Smart Grid is then provided in [31], which provides a detailed review of existing research on selected artificial intelligence techniques applied to load forecasting, power grid stability assessment, fault detection, and security issues in Smart Grids and power systems. One of the important and researched issues is the applicability of blockchain for providing cyber security in different types of networks, including the IoT world, where Smart Grid can be partially included. In [32], the authors provide an overview of blockchains, blockchain structure, consensus algorithms, etc. Furthermore, they compare the algorithms based on their utility and limitations, including other challenges associated with blockchain such as scalability, reliability, interoperability, privacy, and consensus mechanisms for integration with AI, IoT and edge computing. The issues and advances in Deep Learning and Machine Learning with a focus on IoT, industrial networks, and Smart Grid networks are addressed by Alrowais, F. et al. in [33]: Intelligent Intrusion Detection Using Arithmetic Optimization-Enabled Density-Based Clustering with Deep Learning, and Figueiredo, J. in [34]: Deep Learning Model Transposition for Network Intrusion Detection Systems, where the authors propose a model for providing cyber security using deep learning and machine learning. A comprehensive proposal based on Machine Learning models and algorithms was then provided by Rabie, O.B.J. et al. in their paper [35]: “A Proficient ZESO-DRKFC Model for Smart Grid SCADA Security”. A comprehensive overview in the form of a review was then provided by Mazhar, T. et al. in their paper [36]: “Analysis of Challenges and Solutions of IoT in Smart Grids Using AI and Machine Learning Techniques: A Review”, where, among other things, he states that with the help of machine learning, challenging tasks can be handled quite independently. In Smart Grids, computers, and mobile devices indoor temperature control, monitoring of no-safety, and performing routine maintenance can be facilitated. The Internet of Things (IoT) is used to connect the various components of smart buildings. As the IoT concept expands, SGs are being integrated into larger networks. IoT is an important part of SGs because it provides services that improve everyone’s life. Current life support systems were found to be safe and effective in sustaining life. The main objective of the present research is then to find out the motivation for installing IoT devices in smart buildings and networks [36].

The possibility of using neural networks and artificial intelligence for cyber security of power distribution opens a new area and specific applications. This represents an innovative and forward-looking approach to protecting infrastructure and data in the energy sector. These technologies have great potential in improved methods of detection, prevention, and rapid response to cyber threats that could compromise the security and reliability of the distribution network. It can be used for anomaly detection, where neural networks can analyze large volumes of data from the distribution network and identify sudden changes or unusual traffic patterns, indicating possible cyberattacks or undesirable behavior. These anomalies can then be evaluated and addressed.

As part of a comprehensive approach to addressing cyber security in the smart grid, it is then necessary to consider the technical specifics of the technologies and devices used, as described, for example, in “Enhancing Modbus-RTU Communications for Smart Metering in Building Energy Management Systems” [37]. The possibility of using neural networks is then discussed, taking into account the overall reliability of power networks as, e.g., provided by L. Xiao in his article: “Construction Technology and Quality Control of Power and Electrical Engineering Based on Convolutional Neural Network” [38], where Xiao demonstrates that learning using a convolutional neural network, unsupervised analysis of related data and symptom extraction, the effect of controlling quality problems in the development and production process is achieved with efficiency exceeding 71% and an increase of 37% in the overall quality control of projects.

Machine learning and deep learning are subfields of artificial intelligence that are successfully applied to solve cyber security problems. These techniques can be used to develop effective intrusion detection systems that protect data from malicious behavior. Machine learning and deep learning can be applied to various network implementations, applications, algorithms, learning approaches, and datasets to develop a functional intrusion detection system. Some of the computer security problems that can be addressed by deep learning applications include security-oriented program analysis, defending against return-oriented programming (ROP) attacks, achieving control flow integrity (CFI), defending against network attacks, malware classification, anomaly detection based on system events, memory forensics, and fuzzing for software security. In summary, machine learning and deep learning can make a significant contribution to cyber security by continuously analyzing data to find patterns and detect threats. This area was also the subject of significant recent research work. In the field of cyber security, it is worth mentioning the book: Deep Learning Applications for Cyber Security [39], which presents the potential applications of Machine Learning and Deep Learning in the field of cyber security within its twelve chapters. Similarly, the use of these technologies and procedures is also reported in [40]. Machine Learning and Deep Learning have high potential applicability in Methods for Intrusion Detection Systems, which is addressed in [41], and also for the IoT domain, as reported in [42,43]. Interesting research is also presented in [44], where the authors discuss the Machine Learning (ML) and Deep Learning (DL) techniques that are usable to create a secure environment and also discuss various cyber security threats. The use of ML and DL in Industrial Control Systems, with respect to providing cyber security requirements, is discussed by the authors in [45], where anomaly detection in ICS, based on an artificial intelligence algorithm, is presented. ML and DL methods are also applicable for more coherent detection and especially in malware, as reported in [46]. This is stated in [47], among others, in their review. The rationale for addressing Smart Grid cyber security is mainly due to the requirement of maintaining a stable and predictable power delivery service. These problems are then addressed by many researchers, where we can mention [48], who presented a dynamic prediction of retail electricity prices in the Smart Grid based on the Stackelberg model. Then, [49], where the authors discuss the possibility of using ML and DL in refining the power generation forecast, and similarly [50,51,52] discuss the stability and security of the Smart Grid concerning short-term load. In conclusion, Smart Grids are rapidly replacing conventional grids on a global scale. The Smart Grid has its drawbacks, just like any other new technology. A cyberattack on a Smart Grid is one of the most challenging things to stop. The biggest problem is caused by the millions of sensors that are constantly sending and receiving data packets over the network. Cyberattacks can compromise the reliability, availability, and privacy of the smart grid. Users, the communication network of smart devices and sensors, and network administrators are the three layers of an innovative grid network vulnerable to cyberattacks. In [53], the authors discuss the risks and vulnerabilities that can affect the security of critical, innovative grid components. To protect against these dangers, they then present security solutions using various ML and DL-based methods.

The utilization of artificial intelligence represents mainly neural networks, machine learning, and deep learning. Their key advantages, with respect to the problem at hand, include:

- Anomaly detection: machine learning algorithms can analyze large volumes of data to identify patterns and detect anomalies. In the context of security, this can be used to detect unusual or suspicious behavior such as network intrusions, fraud, or malware.
- Intrusion detection and prevention: Machine learning techniques can be used to create robust intrusion detection and prevention systems. By training models on historical data, they can learn to recognize patterns associated with different types of attacks and alert security personnel or automatically block suspicious activity.
- Malware detection: Deep learning models, particularly convolutional neural networks (CNNs), have proven to be very effective in detecting and classifying malware. By learning from large datasets of known malware samples, these models can identify new and emerging threats based on their characteristics and behavior.
- User authentication: machine learning can improve user authentication mechanisms by analyzing various factors such as keystroke dynamics, biometrics, or behavioral patterns. These models can learn to distinguish between genuine users and imposters, which helps prevent unauthorized access.
- Threat detection: machine learning can help analyze vast amounts of security data, including threat feeds, vulnerability databases and security reports. By processing this information, machine learning models can provide valuable insights into emerging threats, identify patterns and help security teams proactively respond to potential risks.
- Predictive analytics: by leveraging historical data and applying machine learning algorithms, security professionals can develop predictive models that anticipate potential security breaches or vulnerabilities. This allows proactive measures to be taken, such as patching systems or strengthening defenses in advance.
- Cyber security automation: machine learning can automate several security tasks such as log analysis, event correlation, and incident response. This reduces the burden on security personnel and enables faster detection and response to security incidents.
- Fraud detection: Machine learning techniques can be used to identify fraudulent activity in a variety of areas, including financial transactions, e-commerce, and online advertising. By learning from past fraud patterns, models can detect suspicious behavior and flag potentially fraudulent activity in real-time.

It’s important to note that while the use of artificial intelligence can greatly enhance security, they are not a silver bullet and should be used in conjunction with other security measures for comprehensive protection. Additionally, these models need to be regularly updated and retrained to adapt with evolving threats and maintain their effectiveness.

## 3. Substation Architecture Model

**Figure 3:**
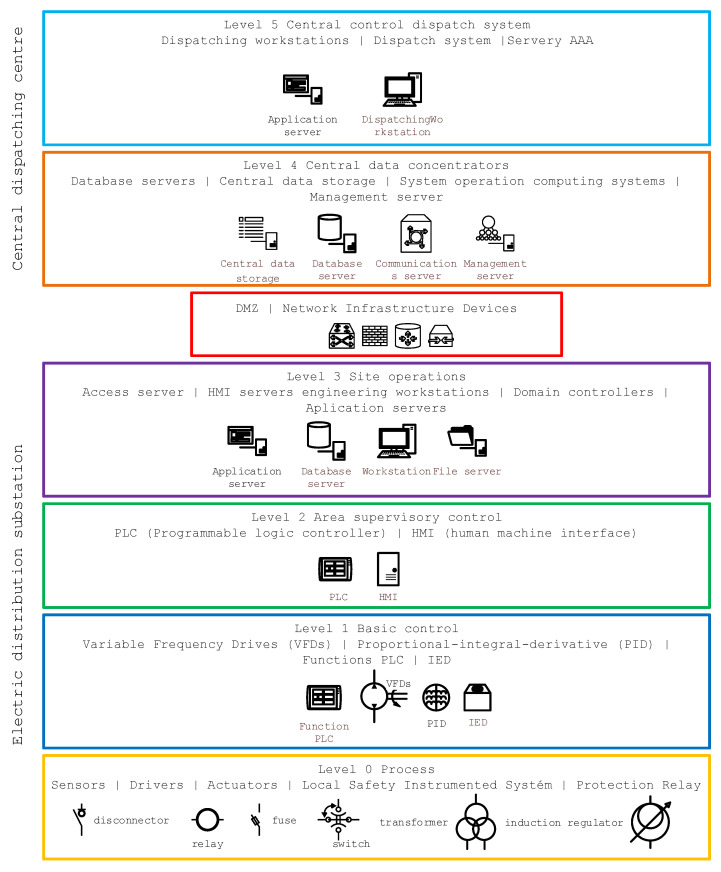
Modified Purdue model of the electrical substation system.

In the first phase, it was necessary to select a suitable model for the general architecture of the technical asset structure of the substation control systems. For the purposes of the project, a model based on the MITRE ATT&CK for Industrial Control Systems (ICS) knowledge base [54] was chosen, supplemented by a layer providing communication with the ICS data hub, typically represented by the data concentrators and the dispatch control system of the TSO, as it is standardly presented in the form of the Purdue model [55] used for industrial systems (Figure 3). This model, despite its “age”, is still used, especially in connection with industrial security, as reported by C. Few [56] in “A case study in the use of attack graphs for predicting the security of cyber physical systems”, or also addressed by [57] in his paper dealing with the risks associated with the rapid development of electric vehicle power devices and their integration into traditional industrial control systems. On top of this model, relevant attack techniques were evaluated answering the question of HOW the attack is conducted, while implementing defined tactics, i.e., WHY the attack is conducted, based on which mitigation measures related to defined technical assets are proposed.

Level 0—Process bus control: This is the level at which the sensors and actuators that are controlled and monitored are located. Level 0 is where the actual control and management process is implemented, and it is essential that all operations take place in real time and without disruptions that could cause problems throughout the control chain.

Level 1—Basic control: Level 1 houses all primary control equipment. Their main purpose is the basic control, operation, and management of level 0 actuators. Typically, Level 1 contains PLCs, variable frequency drives (VFDs), special proportional-integral-derivative units designed for traffic control, (PID) controllers, programmable logic controllers (PLCs), remote terminal units (RTUs), and protection (IEDs).

Level 2—Supervisory control: Many of the functions and systems at Level 2 are the same as those at Level 3, but they are focused on a smaller part or area of the controlled system (e.g., individual fields of a substation). At this level, specific parts of the system are monitored and controlled by local HMI systems, supervisory control systems, I/O servers, engineering workstations, etc. Level 2 is focused on supervisory control LANs, including functions involved in monitoring and controlling physical assets.

Level 3—Operations control: Level 3 contains the systems that support the control and monitoring functions of the entire substation system. These are primarily centralized HMIs and central workstations that provide an overview of all systems that control processes within the substation. Level 3 is where information is passed between the OT layer and the IT systems that provide communications to higher layers of control, primarily the central control system. Systems typically found in Level 3 include, but are not limited to, database servers, application servers (web and reporting), file servers, Microsoft domain controllers, engineering workstations, etc. This level is used to provide communication and interconnection between the LAN (level 2) and central control systems, e.g., data concentrators.

### 3.1. Typical Technical Assets

The following is a brief description of the key technical type assets over which the analysis, design and verification of mitigation measures to reduce the threats, and thus, the resulting risks were carried out.

Control Server—it is a device that functions as both a server and a controller, which hosts the control software used to communicate with lower-level control devices in the ICS network (e.g., remote terminal units (RTUs) and programmable logic controllers (PLCs)). The control server may be referred to in the SCADA system by the terms: MTU, supervisory controller, or SCADA server.

Data Historian—are centralized databases located on a computer installed in the DMZ of the control system, supporting external access to company user data for archiving and analysis purposes using statistical process control and other expert techniques.

Engineering Workstation—for engineering workplaces; highly reliable computing platforms are usually used for configuration, maintenance, and diagnostics of control system applications including other technical assets cooperating with the control system. The computing system typically consists of redundant hard drives, a high-speed network interface, reliable CPUs, powerful graphics hardware, and applications that provide configuration and monitoring tools to perform control system application development, compilation, and distribution of system modifications. Many engineering workstations are implemented using laptops, which, due to their mobile nature, lack of desktop standards, and frequent connection to control system devices and the network, can be used by hackers as entry points for cyberattacks.

Field Controller/RTU/PLC/IED—Controller terminology varies depending on the types of systems that provide specific processing capabilities. Controllers, sometimes referred to as Remote Terminal Units (RTUs) and Programmable Logic Controllers (PLCs), are computerized control units, typically housed in a rack or panel with modular processors and interconnect cards. The units are connected to process equipment and interface via input and output modules to various sensors and controlled devices. Most use a programmable logic-based application that provides state matching and data writing to and from the I/O modules. The interface communicates with the control system communications network through a variety of communication methods and protocols, including serial and network communications. PLCs are typically programmed in the IEC 61131 programming language and are designed for real-time use in an industrial environment. PLCs connected to sensors and actuators are categorized by the number and type of I/O ports and by their I/O scan rates. RTUs are special field devices that support communication with a remote SCADA station or control system with wired and wireless capabilities to communicate with a higher-level controller.

Human–machine Interface (HMI)—An interface called a human–machine interface (HMI) uses graphical, textual, and audio information to present a program to the user (operator) using computer monitors, audio subsystems, and control sequences (e.g., computer keyboard keystrokes, computer mouse movements, and touch screen selections) that the user uses to operate the program. Currently, the following types of HMIs are the most common:

- graphical user interfaces (GUIs) accept input through devices such as a computer keyboard and mouse and provide graphical output on a computer monitor.
- Web user interfaces receive input and provide output by generating web pages that are transmitted over the network and viewed by the user using a web browser. The operator must be able to control the system and assess the state of the system.

The system can expose multiple user interfaces to efficiently utilize a multi-user system defined by individual user permissions. The user interface screens may be optimized to provide appropriate information and control interfaces to operational users, technical users, and data management users.

In many cases, these include video screens or computer terminals, buttons, audio feedback, etc. However, the HMI also provides the means for:

- An input that allows users to control devices (actuators, RTUs, etc.),
- Output that provides output to the user.

Input/Output Server—The Input/Output (I/O) server provides the network interface between control system applications and the end technical devices (located in individual fields) monitored and controlled by the control system applications. The I/O server, also sometimes referred to as a Front-End Processor (FEP) or Data Acquisition Server (DAS), converts control system data into packets that are transmitted over various types of communication media to designated end devices. The I/O server also provides the conversion of data received from various end devices over different communication media into data formatted for communication with control system applications.

Network Infrastructure Devices—It is a communication infrastructure that uses mainly active and passive communication elements (switch, router) with implemented security functionalities such as ACL (Access list, firewall), implements logical separation of VLANs, as well as secure remote access, e.g., by implementing IPSec. Appropriate design of the network infrastructure architecture ensures adequate redundancy and availability of individual technical assets providing the possibility of controlling the control systems of the stations.

Safety Instrumented System/Protection Relay—The Safety Instrumented System (SIS) implements an automatic action to maintain the control system in a safe state or to bring it to a safe state when abnormal conditions are identified. The SIS may implement one or more functions to protect against various system risks within station control systems impacting the state of the transmission and distribution system as a whole. The protective relay function can be used to implement immediate disconnection of dependent components from power system operation caused by a short circuit or other abnormal operation that could result in damage or other disruption to other parts of the power system.

Typical software assets—In the area of software, it is typical for substation automation systems that the actual control software is a proprietary solution of a specific supplier. Individual sub-typical technical assets run on real-time operating systems such as FreeRTOS, VxWorks, QNX, Integrity, ThreadX, Nucleus RTOS, Windows IoT, etc. These systems are also proprietary or use Linux kernels most often based on top of BSD or GNU GPL stage licenses and their modifications. Windows systems, which are based on the monolithic kernel in the embedded variant, are also a significant part of this. Firmware solutions that are developed by manufacturers to meet the specific needs of single-user assets are also an integral part of the software that affects security.

### 3.2. Risks and Threats

**Figure 4:**
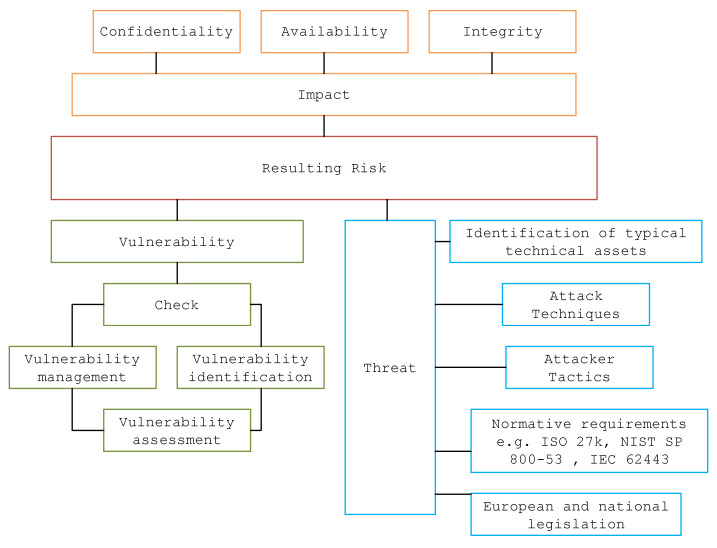
Model of parameters influencing risks.

Determining threats relevant to specific systems is a non-trivial analytical activity, which is influenced by many factors that take into account the specifics of the type of technical assets, tactics and techniques used by attackers on ICS systems, normative requirements in the field of information security and national legislative requirements determined by the national information and cyber security authority. As shown in Figure 4 below, the threat level is one of the significant parameters affecting the resulting risks.

Taking into account the techniques and tactics used by attackers, the MITRE ATT&CK Matrix model [58] is used for ICS systems. This is a general framework that, for the studied environment of ICS systems and their implementation in the Czech Republic, is necessary to keep in mind for which techniques are not relevant for this specific environment. In the analysis, it was found that, for the techniques to activate firmware update, mode module firmware, and system firmware, it is necessary to take into account devices operating in control system networks, where the main monitored parameter availability is evaluated based on data about their reliability and stability. For this reason, changes to firmware or system software are carried out by operators of control systems of electricity substations in a planned manner and subject to a thorough and pre-agreed range of tests on a development copy of the production system, and for this reason, these systems do not run automatic firmware updates, etc. The main threat that can be found in this area is supply chain attacks associated with malware ‘typo squatting’ attacks, which are then exploited for threats against data implemented in the form of ransomware attacks. These techniques are normally eliminated by organizational measures. For the technique of data from information repositories within the implementations of control systems of electrical substations in the Czech Republic, an important element is that external data repositories, which may potentially contain sensitive data, are not operated within existing implementations. This technique is highly important from the point of view of the administrators and operators of these systems, including external contractors and service companies and their IT systems. The importance and relevance of the technique exploits public-facing applications and internet accessible devices is to consider that internet-facing software is not commonly operated within the control systems of electricity substations. Such systems are operated at higher layers within various corporate services. Similarly, Internet-accessible devices are not operated within the control room systems. Within existing implementations, the spear-phishing attachment technique is significantly limited, as email clients that could directly open an infected attachment are not currently operated within the control systems of electrical substations. This technique is highly relevant in terms of protecting any IT or OT system, but its misuse must be prevented in a different part of the operator’s, administrator’s, supplier’s, or service organization’s information system than the control station. According to ISO 27001 and 27002, the issues of using supply chain compromise techniques concerning the compromise of systems within the supply chain are already addressed by the administrators and operators of these systems during the preparation and implementation phase of projects. An important technique is weak encryption, where it is important to note that encryption is a specific weakness of most Level 0 and Level 1 devices, according to Figure 1. In particular, combined IEDs, whose main function is the electrical protection of primary substation equipment, are not equipped today with sufficient hardware capacity to encrypt communications. Therefore, the implementation of any encrypted communications must be implemented between Level 2 devices and above. The attacks associated with exploiting vulnerabilities and low level of cryptographic protection, which are clearly threats against data and availability, are also mentioned in the ENISA threat landscape 2022 study.

## 4. Security Baseline Proposal

The objective of establishing a Security Baseline for station energy management systems is to reduce the resulting risks associated with the operation and management of these systems and the associated services they provide. The risk, the value of which is determined according to [59], is shown in Formula (1).

Risk = impact × vulnerability × threat(1)

According to Figure 4, impact is perceived as the value of an information asset, and its value is most often determined according to the relationship (2). Impact of single assets expresses the value of data on single assets concerning their availability, confidentiality and integrity, i.e., the basic characteristics describing the value of the data or the assets on which the data are located and processed.

Impact = max (confidentiality; availability; integrity)(2)

The determination of the vulnerability value is technically determined by the value set in the National vulnerability database, whose value can be obtained directly from the vulnerability database or calculated using the official Common Vulnerability Scoring System Version 3.1 Calculator [60]. The calculation of the vulnerability value itself is very complex and in the basic classification, it is calculated as function of the Impact and Exploitability sub score equations, where the Base score is defined as Formulas (3)–(6):

If (Impact sub score ≤ 0) 0 else  Scope Unchanged − Roundup(Minimum [(Impact + Exploitability), 10]) 
Scope Changed − Roundup(Minimum [1.08 × (Impact + Exploitability), 10])(3)

and the Impact sub score (ISC) is defined as,

Scope Unchanged 6.42 × ISC_Base_

Scope Changed 7.52 × [ISC_Base_ − 0.029] − 3.25 × [ISC_Base_ − 0.02]^15^ (4)

where,

ISC_Base_ = 1 − [(1 − Impact_Conf_) × (1 − Impact_Integ_) × (1 − Impact_Avail_) (5)

and the Exploitability sub score is,

8.22 × AttackVector × AttackComplexity × PrivilegeRequired × UserInteraction(6)

The final parameter that affects the resulting risk is the level of threat, i.e., the facts, events, forces or persons whose action (activity) may cause damage, destruction, and the loss of confidence or value of the asset. Threats that are relative to the power station control systems domain are defined based on the areas that affect the value of the threat, as shown in Figure 4, and are driven by the frequency of threat occurrence and the opportunity to exploit the threat. To establish a security baseline for station energy control systems, considering that these systems are regulated not only from a security perspective but also in relation to the service provided, they must meet a range of technical and security standards. To establish the security baseline, the standards were EN IEC 62443-3-3 [61], EN IEC 62443-4-2 [62], and EN ISO/IEC 27001 [63]. The generalized risks were then determined as the fulfillment of threats of non-fulfillment of the requirements specified in these standards. The design of the Vulnerable Security baseline then considers the results of the analysis of selected Attack Techniques that can exploit known vulnerabilities and, thus, activate the associated threats.

### 4.1. Excluded Areas of ISO 27001

In relation to ČSN EN ISO/IEC 27001, the solution did not include area A7 Human Resources Security, because this area is fully in the responsibility of the system operator or supplier and concerns its internal rules of human resources management. Another excluded area is A16 Information Security Incident Management, because this area is addressed at higher levels of the Purdue model, most often at the cluster level in the form of technological, operational, and security oversight. The addressed issue of power station control systems is intended to provide the required information, but no specific oversight is implemented at the addressed levels. The sub-forest exempted area is A17—Information security aspects of managing the continuity of an organization’s activities. This area is relevant to the whole information security assurance complex, where the security measures specified are binding on the whole organization and binding on the areas to the extent determined by the organization itself.

### 4.2. Security Baseline for Power Distribution Control Systems

This chapter proposes mitigation measures to reduce the risk of misuse of the above techniques used by attackers on the model technical assets. Individual mitigation measures are further mapped to the requirements arising from NIST SP 800-53 Rev. 4, IEC 62443-3-3:2013, IEC 62443-4-2:2019, ISO 27001 and the national decree 82/2018 Coll., on cyber security (hereafter DoCS), which is binding for energy entities in the Czech Republic and is a national implementation of the requirements of Directive (EU) 2016/1148 of the European parliament and of the council of 6 July 2016, concerning measures for a high-common level of security of network and information systems across the Union. Therefore, the individual mitigation measures clearly contribute not only to ensuring an adequate level of cyber security, but also to meeting legislative and normative requirements. Table 2 below presents a summary of the technical measures with an overlap into organizational measures, which must be implemented without undue delay and their implementation will ensure minimum compliance with the technical requirements arising from the legislation, with the exception of §23 and §24 of Decree 82/2018 Coll., which deal with the detection of cyber security events and the collection and evaluation of cyber security events, respectively, which are addressed in the supplementary mitigation measures and recommendations.

Table 3 below presents a set of recommended mitigations to protect against attackers’ techniques. These mitigation measures are mainly technical in nature and, for their effectiveness, assume the implementation of organizational measures listed in Decree 82/2018 Coll. on Cyber Security; in particular, the implementation of an information security management system according to §3, §4 asset management and §5 risk management. It is also necessary to have rules in place for the management of contractors within the scope of the requirements defined in §8 to ensure the integrity of human resources within the framework specified in §9.

The above set of mitigation measures, which form a comprehensive Security baseline for energy management systems, were verified in two phases in cooperation with The National Cyber and Information Security Agency (NCIS), which is the central administrative authority for cyber security, including the protection of classified information in the field of information and communication systems and cryptographic protection. During this validation phase, the continuity of the uniform mitigation measures with the requirements of national legislation and normative requirements was checked. In the second phase of the validation, the cooperation with the industrial partner Hitachi Energy Czech Republic s.r.o., which is one of the top five most important suppliers of power station control systems in the Czech Republic and the EU, was carried out. The complexity and feasibility of the deployment of uniform measures within a security polygon supplemented with technological elements simulating different versions of operated and supplied power station control systems were tested. Here, it should be stressed that the lifetime of the delivered systems is calculated to be between 10 and 15 years and, therefore, for some mitigation measures, a condition is specified that its feasibility depends on the age of the system in question, as some of the legacy systems contain components that meet the definition of a legacy system. For these components, it is then necessary to increase cyber protection at the outer perimeter of the system. The above outputs of the validation phases of the proposed Security baselines for station power systems are available to the authors and can be provided upon request.

## 5. Conclusions

As already mentioned, the issue of ensuring the cyber security of substation automation systems is a non-trivial matter. Substation automation involves the use of diverse technologies from the traditional IT world, but also industrial technologies. Last, but not least, it is affected by many regulatory and legislative restrictions. In general, approaches to cyber assurance can be divided into organizational and technological levels. Organizational security not only defines the procedures and requirements for ensuring cyber security but, in the form of internal management documents, combines the operational and security perspectives. The implementation of security measures based on technical measures is significantly influenced by the choice of specific technologies. Despite the heterogeneity of technologies used with varying degrees of automation and autonomy, it is important to both standardize the security requirements to the maximum extent possible and meet the safety legislative and normative requirements, while at the same time, ensuring they do not interfere with the fulfillment of technological standards and norms.

It is more than obvious that designing a model or set of measures for a specific combination of technologies significantly reduces its reusability in other projects for substation automation suppliers or power distributors. If we want to methodically support the process of ensuring cyber security for substation automation, there is a possibility of using security baselines with an emphasis on the specifics of the technologies used. The important approach for the creation of a security base line is the analysis of techniques used to breach the cyber security of industrial systems according to the general MITRE knowledge base of mitigation measures, which was implemented to the substation automation area with respect to its specifics. At the same time, the security baseline considers and integrates the requirements of the most important security standards and legislative requirements.

The benefit of the created security baseline lies mainly in the fact that it considers the specifics associated with the industrial technologies used by the suppliers, but also considers the standardized model of the smart substation architecture. Design and implementation of the safety base line is thus a unique tool for ensuring comprehensive safety requirements, while adhering to technological and safety standards. Let us summarize the benefits of this proposal.

- Considers the heterogeneity and propriety of individual solutions according to the supplier, but also maps individual functionalities into a generalized Modified Purdue model of the electrical substation system, simplifying risk analysis and utilization of standard assets.
- Provides the supplier with a comprehensive clear checklist of safety requirements, the fulfillment of which meets national and international safety legislation and safety standards. The specific technical solution is then usable on the global market, and from the supplier’s point of view, it is difficult to standardize it.
- It provides transmission and distribution network operators with clear instructions on how to define security requirements for the supply chain, including solutions for their fulfillment, which has also been approved by the National Agency for Information and Cyber Security, which is responsible for checking the fulfillment of national security requirements.

The paper presented the results of the design of the security of power control systems of stations using the implementation and verification of mitigating scalds that not only reflect cyber threats to ICS systems, but their implementation will ensure compliance not only with information and cyber security standards but also the fulfillment of relevant legal requirements, which are mandatory for these critical systems. Twenty-two basic mitigation measures forming the Security baseline group were proposed and mapped to ensure a minimum mandatory level of security. In addition, another 32 enhancement measures were designed and tested for the so-called Extended Security baseline set, which take into account the expanding attack techniques and increase the resilience of the system as a whole. The proposed solution also includes a minimum level of organizational measures, which are an integral part of the security assurance process, and every supplier and system operator is obliged to implement them within their security documentation. The proposed Security baseline was technically verified within the cyber polygon in cooperation with the technology partner Hitachi Energy Czech Republic s.r.o. on selected types of existing and newly delivered solutions. The mapping of the proposed Security baselines to normative and legislative requirements was performed as specified by the government agency: The National Cyber and Information Security Agency. Security baselines thus become part of the security measures implemented on existing and newly built power station control systems.

## Figures and Tables

**Table 1:** Classification of cyberattacks on the smart grid according to TCP/IP with implications for the CIA triad. Source [17].

Network Layer	Confidentiality	Integrity	Availability
Application Layer	Data-Injection Attack	-	LDoS, HTTP Flooding, Buffer Overflow
Transport Layer	IP-Spoofing, Data-Injection, Sniffing, MITM, Password Pilfering Attacks	Replay, Covert, Wormhole, Data-Injection, MITM, Spoofing Attacks	Wormhole, MITM, Buffer Overflow, Buffer Flooding, DdoS Attacks
MAC Layer	ARP-Spoofing, Traffic Analysis, MITM Attacks	ARP-Spoofing, TSA, MITM Attacks	Spoofing, TSA, Jamming, DdoS, Flooding, MITM Attacks
Physical Layer	Eavesdropping	Smart Meter Tampering Attacks, TSA	Jamming Attacks, TSA

**Table 2:** Urgent technical and organizational mitigation measures.

Id	Mitigating Measures	Description of Measures	NIST SP 800-53 REV. 4	IEC 62443-3-3:2013	IEC 62443-4-2:2019	ISO 27001	DOCS
M_01	Access Management [64]	Technical measures using Access Management technology are used to enforce authorization policies, especially when existing devices in the substation do not provide sufficient capabilities to support user identification and authentication. These technologies are typically used for in-line network devices or gateways to prevent access by unauthenticated users. They are integrated with an authentication service for the initial authentication of user credentials. The measure is directly linked to the organizational measures set out in §12 of the DoCS, the implementation of which is an integral part of a functional Access Management system. Mitigation is the responsibility of the supplier and the configuration engineer. Mitigation covers access to HW, especially SW platforms.	AC-3	SR 2.1	CR 2.1	A.9.1, A.9.2, A.9.3, A.9.4	§12. §19, §20, §25, §26
M_02	Account Use Policies [65]	The technical measure reflects the need to configure functions related to the use of the account, e.g., locking the account for a given number of unsuccessful login attempts, setting specific login times, etc. The measure has a direct link to the organizational measures mentioned in §4, where it is necessary to have asset management in place for relevant settings of Account Use Policies, as well as §10 reflecting traffic and communication management, §12 access control. It is also necessary to explicitly implement this measure in §13 Acquisition, Development, Maintenance based on the establishment of security policies and their transfer to supply and service contracts. Mitigation is the responsibility of the supplier and the configuration engineer. The design must come from the system architect. Mitigation covers access to HW, especially SW platforms.	IA-5	SR 1.11	CR 1.11	A.6.2, A.9.1, A.9.2, A.9.3, A.9.4, A.12.1, A.12.2, A.12.3, A.12.4, A.12.5	§4, §10, §12, §13, §19, §22, §25, §26
M_03	Antivirus/Antimalware [66]	It is a service used to detect malware. In industrial environments, antivirus/antimalware installations should be limited to resources that are not involved in critical or real-time operations to minimize the impact on system availability. All products must first be tested in a representative test environment prior to deployment on production systems. Mitigation is the responsibility of the supplier and the configuration engineer. Mitigation mainly covers protection to SW platforms.	SI-3	SR 3.2	CR 3.2	A.8.3, A.12	§10, §21
M_04	Authorization Enforcement [67]	It is necessary to implement permission management on ICS equipment and systems according to RBAC procedures with emphasis on the allocation of minimum permissions. it is then appropriate to use the IEC 62351 standard in the ICS area for both user and system and operational processes. The design must come from the system architect. Mitigation covers access to HW, especially SW platforms.	AC-3	SR 2.1	CR 2.1	A.8.3, A.9.1, A.9.2, A.9.4, A.12.5	§10, §12, §19, §25, §26, §28
M_05	Code Signing [68]	Using digital signature authentication strengthens binary and application integrity to prevent the execution of untrusted code. The application of this measure must consider the technical limitations of Legacy systems that are in real-life operation and reflect this in the applicability statement, according to §5 of the DoCS. Mitigation is relevant in the supplier-customer relationship. Mitigation covers access to HW (firmware), especially SW platforms.	SI-7	SR 3.4	CR 3.4	A.10.1, A.12.1, A.12.5, A.14.1, A.14.2	§10, §11, §13, §25, §26, §28
M_06	Data Backup [69]	When taking and storing backups of data from end-user systems and critical servers, it is necessary to ensure that backup and storage systems are enhanced to store backups in a separate offsite environment. Plans and responses to potential incidents, including backup image and configuration management for key systems, must be maintained and tested to enable rapid recovery to respond to attacker activity that affects the control, view, or availability of systems. Based on the asset management according to §4 and §5 of the DoCS, a process model per information and technical asset must be established with respect to the recovery plan, data value and configurations. Mitigation mainly covers protection to SW platforms and data. The design must come from the system architect.	CP-9	SR 7.3	CR 7.3	A.6.2, A.12.1, A.12.3	§4, §10, §27, § 28
M_07	Disable or Remove Feature or Program [70]	This service is used to remove or deny access to unnecessary and potentially vulnerable software to prevent abuse by attackers. Only original systems and software directly related to its functions must be run on the substation. Technical implementation must be preceded by a comprehensive §4 asset management process. Mitigation is the responsibility of the supplier and the configuration engineer. Mitigation covers access to HW, especially SW platforms.	CM-7	SR 7.7	CM 7.7	A.8.1, A.9.1, A.9.2, A.9.3, A.9.4	§4, §12, §19, §25, §26
M_08	Execution Prevention [71]	This is used to block the execution of code in ICS by the applications themselves and to block the execution of scripts. It is necessary to control the appropriate permissions with respect to inheritance of the subprocess generation framework. As part of the implementation of this measure, FAT tests shall be performed on the relevant test environment to maintain the full range of services provided, considering the maturity of the system and its dependency on other TR traffic management processes. The design must come from the system architect. Mitigation covers access to HW, especially SW platforms.	SI-3	SR 3.2	CR 3.2	A.9.4	§12, §19, §25
M_09	Exploit Protection [72]	A system for ensuring the detection and blocking of conditions leading to software misuse or abuse. The implementation of the measures shall consider any legacy systems operating in individual TRs and downstream systems. Mitigation is the responsibility of the supplier and the configuration engineer. Mitigation mainly covers protection to SW platforms and data.	SI-16	SR 3.2	CR 3.2	A.8.3, A.9.4, A.12.1, A.12.5, A.14.1	§10, §13, §18, §19, §25, §26
M_10	Limit Hardware Installation [73]	This service shall block users or groups of users from installing or using unapproved hardware on substation systems, including USB devices. If, for example, a hardware key is required to run specialized substation systems, the physical perimeter protection of the, according to §17, must be increased and this solution must be reflected in the PoA. Mitigation is relevant in the supplier-customer relationship. Mitigation covers access to HW (firmware), especially SW platforms.	MP-7	SR 3.2	EDR 3.2	A.11.2, A.12.1, A.14.1	§10, §13, §17, §18, §21
M_11	Mechanical Protection Layers [74]	Physical security is an integral part of cyber protection within layered (onion) protection. It is therefore necessary, from a security perspective, to address the location of the facility itself, its security, surveillance and its zoning, which allows us to effectively divide the individual elements of physical protection according to individual, embedded, access zones. Mitigation is related to the operational environment and is entirely the responsibility of the customer. It mainly covers access to HW platforms.	N/A	N/A	N/A	A.11.2	§17
M_12	Network Allowlists [75]	It is strongly recommended to implement lists of allowed networks and to determine for which connections (e.g., IP address, MAC address, port, protocol) connections can be made. Allowed protocol lists that operate at the application layer (e.g., DNP3, Modbus, HTTP) are described in M_32. The design must come from the system architect. Mitigation covers access to HW, especially SW platforms.	AC-3	N/A	N/A	A.13.1	§18
M_13	Network Segmentation [76]	The network design should include an isolated section for critical systems, functions or resources. Physical and logical segmentation may be used to prevent access to potentially sensitive systems and information. A DMZ or jumped approach may be used to secure the station bus and ensure that the critical assets are not accessible from the internal network. Furthermore, it is necessary to restrict network access to specific systems and services, in addition to securing and preventing systems from other networks from accessing critical management systems. For example, in IEC 62443, systems at the same security level should be grouped into a “zone” and access to this zone is restricted and mechanisms are in place to restrict data flows between zones by segmenting the network. The design must come from the system architect. Mitigation covers access to HW, especially SW platforms.	AC-3	SR 5.1	CR 5.1	A.13.1	§18, §28
M_14	Operating System Configuration [77]	Changes to the underlying operating system configuration are required to strengthen system protection against attackers. In this section, in addition to recommending vendors, the mitigation measures outlined in the AT&T Operating System Configuration Project are used effectively. Mitigation is the responsibility of the supplier and the configuration engineer. Mitigation is relevant in the supplier-customer relationship. Mitigation covers access to SW platforms in particular.	CM-7	SR 7.7	CR 7.7	A.14.1	§18, §28
M_15	Out-of-Band Communications Channel [78]	Introduces alternative methods to support communication requests during communication failures and data integrity attacks. The implementation of this technical measure shall consider, on the basis of a risk analysis, possible warnings from the NCIB concerning technical and software resources. At the same time, the availability requirements for unified systems and data must be clearly identified. The design must come from the system architect. Mitigation covers access to HW, especially SW platforms.	SC-37	N/A	N/A	A.12.1, A.13.1	§10, §11, §13, §18
M_16	Privileged Account Management [79]	Rules for the creation, modification, use and permissions associated with privileged accounts, including the root account, must be established and clearly described. Mitigation is the responsibility of the supplier and the configuration engineer. Mitigation covers access to HW, especially SW platforms.	AC-2	SR 1.3	CR 1.3	A.9.1, A.9.2, A.9.3, A.9.4	§12, §19, §20
M_17	Restrict File and Directory Permissions [80]	Rules for restricting access and setting permissions to directories and files that are not specific to users or privileged accounts must be set and clearly documented. Mitigation is the responsibility of the supplier and the configuration engineer. Mitigation covers access to HW, especially SW platforms.	AC-6	SR 2.1	CR 2.1	A.12.1, A.12.2, A.12.3, A.12.4	§10, §11, §13, §20, §25
M_18	Restrict Registry Permissions [81]	Set restrictions on the ability to modify certain sub registers or keys in the MS Windows registry. Here it is recommended to follow the data sources available in the AT&T Windows Registry project area. The unique measures must be tested on an appropriate test environment. Mitigation is the responsibility of the supplier and the configuration engineer. Mitigation covers access to HW, especially SW platforms.	AC-6	SR 2.1	CR 2.1	A.12.1, A.12.2, A.12.3, A.12.4	§10, §11, §13, §25
M_19	Restrict Web-Based Content [82]	It is necessary to implement strict restrictions on access to web services, block downloading/sending files, disable script execution, restrict installation of browser extensions, etc. Mitigation is relevant in the supplier-customer relationship. Mitigation covers access to SW platforms in particular.	SC-18	SR 2.4	HDR 2.4	A.12.1, A.12.2, A.12.3, A.12.4	§10, §11, §13, §25
M_20	Vulnerability Scanning [83]	This measure is used to implement regular vulnerability scanning, which is used to find potentially exploitable software vulnerabilities and leads to their remediation. The implementation of this measure is dependent on the implementation of the §4 asset management and §5 risk management process. One effective solution is to implement a comprehensive configuration database of the system and its downstream processes. Mitigation is the responsibility of the vendor and the system architect. Mitigation is relevant in the supplier-customer relationship. Mitigation covers access to SW platforms in particular.	RA-5	N/A	N/A	A.12.6	§11, §25
M_21	Watchdog Timers [84]	Implementation of Watchdog Timers, in order to provide early indication that a device or system is not responding. Mitigation covers not only access in HW but especially to SW platforms. The design must come from the system architect.	N/A	N/A	CR 7.2	A.13.1	§27
M_22	Credential Access Protection	Some network devices are capable of storing passwords for local accounts, either in plain text or encrypted format. It is important to ensure that where technically possible, local passwords are always encrypted. Mitigation is the responsibility of the supplier and the configuration engineer. Mitigation covers access to HW, especially SW platforms.	IA-5	SR 1.5	CR 1.5	A.10.1	§18, §26

**Table 3:** Additional technical and organizational measures.

Id	Mitigating Measures	Description of Measures	NIST SP 800-53 REV. 4	IEC 62443-3-3:2013	IEC 62443-4-2:2019	ISO 27001	DOCS
M_23	Active Directory Configuration [85]	When configuring Active Directory services, make sure that security identifier (SID) filtering, etc., is also used. The implementation of this technical support is significantly affected by the age of the deployed system. The implementation of this measure then supports the requirements specified in §4, §10 and §12. Its requirements need to be taken into account in the implementation of the §13 acquisition, development and maintenance principle. Mitigation is the responsibility of the vendor and the configuration engineer in necessary coordination with the system architect. Mitigation mainly covers protection to SW platforms and data.	N/A	N/A	N/A	A.6.2, A.8.1, A.8.3, A.9.2, A.9.4, A.12.1, A.12.4	§4, §10, §12, §13, §19, §25, §26, §28
M_24	Application Developer Guidance [86]	This is the documented provision of specific guidance and training to application developers to avoid implementing security weaknesses in software development that could be exploited by an attacker. These rules should be part of defined vendor relationships and their management. Mitigation is the responsibility of the vendor and the configuration engineer in necessary coordination with the system architect. Mitigation covers access to HW, especially SW platforms, including links to the continuity and recovery lanes.	AT-3	N/A	N/A	A.15.5, A.14.1, A.14.2, A.14.3	§8, §10, §11, §13, §25, §28
M_25	Application Isolation and Sandboxing [87]	Restricts code execution on virtual environments or when transferring to end systems. It is recommended that this principle is also implemented in the maintenance services provided and in the transfer of all data between the public and internal communication infrastructure. In the substation area, these are physically separate data circuits, where any direct connection to other data circuits is not allowed. Mitigation is the responsibility of the vendor and the configuration engineer in necessary coordination with the system architect. Mitigation mainly covers protection to SW platforms and data.	SI-3	SR 5.4	CR 5.4	A.9.4, A.12.1, A.12.5, A.14.2	§10, §11. §13, §19, §25, §26. §28
M_26	Audit [88]	To identify potential weaknesses, it is necessary to conduct audits and scans of systems, permissions, insecure software, insecure configurations, etc. Documented periodic device integrity checks, verifying the correctness of firmware, software, programs and configurations must be implemented. Integrity checks, which typically include cryptographic hashes or digital signatures, should be compared against checks obtained against known valid states, especially after events such as device reboots, program downloads, or program restarts. An audit should be implemented by both: the substation technology vendor and the substation operator. The possibilities of transferring audit outputs between stakeholders should be considered in contractual arrangements. Mitigation is intended for the customer side and its security and IT department. Both processes and HW and SW configurations are audited.	SI-7	SR 3.4	CR 3.4	A.6.2, A.8.3, A.9.2, A.10.1, A.12.1, A.12.5, A.13.1, A.18.1, A.18.2	§3, §10, §12, §16, §19, §24, §25, §26, §28, §30
M_27	Boot Integrity [89]	The technical measure is based on the principle of using a secure method to boot the system and verify the integrity of the operating system and its loading mechanisms. The deployment of this technical measure is significantly dependent on the age of the compared systems. Mitigation is the responsibility of the vendor and the configuration engineer in necessary coordination with the system architect. Mitigation covers access to HW, especially SW platforms.	SI-7	N/A	CR 3.14	A.10.1, A.12.2, A.12.4, A.12.5, A.14.1, A.14.2, A.18.2	§3, §10, §13, §16, §26, §21, §22, §25, §28
M_28	Communication Authenticity [90]	When communicating over an untrusted network, it is necessary to use secure network protocols that authenticate the sender of the message and can verify its integrity. This can be done through 802.1x or digital signatures. The solution can detect spoofed network messages and unauthorized connections. The design must come from the system architect. Mitigation mainly covers protection to SW platforms and data.	SC-8; SC-23	SR 3.1	CR 3.1	A.13.1	§18
M_29	Data Loss Prevention [91]	Data Loss Prevention (DLP) technology is commonly deployed to protect technology and technical data and information relevant to can generally be used to identify hostile attempts to penetrate operational information such as technical plans, trade secrets, configurations, intellectual property or process telemetry. DLP features can be built into other security products such as firewalls or standalone suites running as agents on the network. The DLP feature can be configured to prevent the transmission of information through corporate sources such as email, the web, and physical media such as USB in the case of hosted solutions. The design must come from the system architect. Mitigation mainly covers protection to SW platforms and data.	N/A	SR 4.1	CR 4.1	A.6.1, A.6.2, A.9.2, A.12.4	§10, §11, §13, §12, §19, §22
M_30	Encrypt Network Traffic [92]	Strong cryptographic techniques and protocols must be used to prevent eavesdropping on network communications. A list of approved cryptographic devices is available on the website The National Cyber and Information Security Agency (NÚKIB) based on the recommendations of The National Institute of Standards and Technology (NIST) technical laboratory in the field of cryptography. The design must come from the system architect. Mitigation mainly covers protection to SW platforms and data.	SC-8	SR 4.1	CR 4.1	A.10.1, A.13.1	§18, §26
M_31	Encrypt Sensitive Information [93]	This involves ensuring the implementation of strong encryption to protect stored data. The design must come from the system architect. Mitigation mainly covers protection to SW platforms and data.	SC-28	SR 4.1	CR 4.1	A.10.1	§18
M_32	Filter Network Traffic [94]	In ICS networks it is necessary to implement filtering of incoming and outgoing traffic based on the implementation rules for filtering. It is necessary to define the allowed traffic using whitelists, both at packet, state and application firewall level. It is also generally recommended to also deploy deep packet inspection, specific firewalls for SCADA automation/protocol. The design must come from the system architect. Mitigation mainly covers protection to SW platforms and data.	AC-3; SC-7	SR 5.1	CR 5.1	A.13.1	§18
M_33	Human User Authentication [95]	Access to system and technical resources of ICS systems must be controlled at the level of user authentication and authorization. It is often problematic or technically impossible to deploy multi-factor authentication within ICS systems. Therefore, it is necessary to strengthen the perimeter of protection accordingly, e.g., to implement multi-factor authentication at the physical entrance to ICS resources, to implement account management and account permissions, to increase the frequency of technical and process audits, etc. Mitigation is the responsibility of the vendor and the configuration engineer in necessary coordination with the system architect. Mitigation is related to the operational environment and is entirely the responsibility of the customer. It mainly covers access to HW platforms.	IA-2	SR 1.1	CR 1.1	A.9.1, A.9.2, A.9.3, A.9.4	§12, §19, §25, §26
M_34	Limit Access to Resource Over Network [96]	Mitigation is based on restricting access to shared system and technical resources. In order to exploit remote accesses, secure protocols, appropriate cryptographic protection, and a tightened lottery of these activities must be uniquely described, implemented, and enforced. The design must come from the system architect. Mitigation mainly covers protection to SW platforms and data.	AC-3; SC-7	SR 5.1	CR 5.1	A.13.1	§18, §20
M_35	Minimize Wireless Signal Propagation [97]	By its physical nature, a wireless signal propagates outside the boundaries of an organization, providing attackers with the ability to monitor traffic on that network and attempt to gain unauthorized access to it. When it is necessary to use a Wi-Fi network in the environment of transformation stations (it is generally not recommended or prohibited), it is essential that appropriate measures are put in place to detect and limit unnecessary Wi-Fi propagation. At the same time, this must be considered in the actual design of the network and wireless technologies must not be used for the transmission of control and monitoring data. The design must come from the system architect. Mitigation mainly covers protection to SW platforms and data.	SC-40	SR 1.6	CR 1.6	A.13.1	§18
M_36	Mitigation Limited or Not Effective [98]	This service is based on the principle of abusing the given systems functions. Mitigation is possible by preventive checks and maintenance of the systems. The solution is linked to the implementation of measures M_20, M_26 and M_50. The design must come from the system architect. Mitigation mainly covers protection to SW platforms and data.	N/A	N/A	N/A	A.14.1	§13
M_37	Multi-factor Authentication [99]	The service uses two or more factors to authenticate for system access. For example, a username and password supplemented by a token from a physical smart card or token generator. In industrial control systems, assets such as IDEs, workstations, and HMIs have high requirements for operational control and real-time security that can limit the use of multi-factor control. Deployment is affected by the age of the system. Mitigation is the responsibility of the supplier and the configuration engineer. The design must come from the system architect. Mitigation covers not only access in HW but especially to SW platforms.	IA-2	SR 1.7	CR 1.7	A.6.2, A.9.4	§10, §19, §25, §26
M_38	Network Intrusion Prevention [100]	It is recommended to use signatures to detect network traffic intrusions and block unauthorized traffic at the network boundaries. In industrial control systems, network intrusion prevention should be configured so that it does not interfere with protocols and communications responsible for real-time control or security-related functions. The design must come from the system architect. Mitigation mainly covers protection to SW platforms and data.	SI-4	SR 6.2	CR 6.2	A.13.1	§18
M_39	Operational Information Confidentiality [101]	Implements/introduces a mechanism to protect the confidentiality of information related to operational processes, facility locations, facility configurations, programs, or databases that may contain information usable to derive organizational trade secrets, processes, and other intellectual property. The design must come from the system architect. Mitigation mainly covers protection to SW platforms and data.	N/A	SR 4.1	CR 4.1	A.6.1	§11, §13
M_40	Password Policies [102]	It is necessary to describe and implement password security rules with an emphasis on enforcing secure password policies according to user persistence. This also applies to system process accounts. Mitigation is the responsibility of the supplier and the configuration engineer. The design must come from the system architect. Mitigation covers access to HW, especially SW platforms.	IA-5	SR 1.5	CR 1.5	A.9.1, A.9.2, A.9.3, A.9.4	§12, §19
M_41	Redundancy of Service [103]	The design and implementation of the logical and physical topology of the network within the electrical substations must consider the availability requirements of the services provided and thus ideally be fully redundant, Minimal redundancy is required for critical services of the entire system. Mitigation is the responsibility of the vendor and the configuration engineer in necessary coordination with the system architect. Mitigation covers access to HW, especially SW platforms., including links to the continuity and recovery lanes.	CP-9	N/A	N/A	A.12.1, A.12.2, A.12.3, A.12.4	§10, §11, §13, §27, §28
M_42	Restrict Library Loading [104]	The mitigation measure is aimed at ensuring the integrity of the libraries used within the operating system and unified applications. For these parts, an appropriate level of integrity must be ensured. This can be effectively achieved by using verified resources and libraries. Thus, in an ICS environment, even generally reliable sources such as GitHub cannot be used without prior vetting and assurance of integrity. Mitigation is the responsibility of the supplier and the configuration engineer. The design must come from the system architect. Mitigation covers access to HW, especially SW platforms.	CP-7	SR 7.7		A.12.1, A.12.2, A.12.3, A.12.4	§10, §11, §13, §25
M_43	Safety Instrumented Systems [105]	Utilize Safety Instrumented Systems (SIS) as an additional layer of protection for safety scenarios that may cause property damage. An SIS typically includes sensors, logic controllers, and a final control element that can be used to automatically respond to an unsafe condition. It is necessary to ensure that all SIS are segmented from the operational networks. Mitigation is the responsibility of the supplier and the configuration engineer. The design must come from the system architect. Mitigation mainly covers protection to SW platforms and data.	N/A	N/A	N/A	A.13.1	§18
M_44	Software Configuration [106]	Implements documented procedures for software (non-operating system) configuration changes to mitigate security risks associated with software operation. Mitigation is the responsibility of the supplier and the configuration engineer. The design must come from the system architect. Mitigation mainly covers protection to SW platforms and data.	CM-7	SR 7.7	CR 7.7	A.12.1, A.12.2, A.12.3, A.12.4	§10, §11, §13, §22, §28
M_45	Software Process and Device Authentication [107]	Devices that connect remotely to other systems should require strong authentication to prevent unauthorized communication. Among other things, software processes should also require authentication when accessing APIs. If necessary, authentication of devices and software processes in the ICS environment should be required. Mitigation is the responsibility of the supplier and the configuration engineer. The design must come from the system architect. Mitigation mainly covers protection to SW platforms and data.	IA-9	SR 1.2	CR 1.2	A.12.1, A.12.2, A.12.3, A.12.4	§10, §11, §13, §19, §18, §25, §28
M_46	SSL/TLS Inspection [108]	In the three cases where it is necessary for electrical substation systems to communicate to an Internet-type network, it is imperative that they have implemented TLS (or its predecessor SSL) for the purpose of encrypted communication and thus ensuring its confidentiality. The design must come from the system architect. Mitigation mainly covers protection to SW platforms and data.	N/A	N/A	N/A	A.13.1	§18
M_47	Static Network Configuration [109]	For security reasons, it is recommended to use static network configurations. Protocols that require dynamic discovery/addressing (e.g., ARP, DHCP, DNS) can be used to manipulate network message passing and enable various MitM attacks. This measure may not always be applicable due to device limitations or problems posed by different network configurations. The design must come from the system architect. Mitigation mainly covers protection to SW platforms and data.	CM-7	SR 7.7	CR 7.7	A.13.1	§18
M_48	Supply Chain Management [110]	This is purely an organizational measure that is recommended to be implemented as part of a supply chain management program, including policies and procedures to ensure that all equipment and components are sourced from trusted suppliers and tested to verify their integrity. The design must come from the system architect. Mitigation mainly covers protection to SW platforms and data.	SA-12	N/A	N/A	A.14.1	§8
M_49	Threat Intelligence Program [111]	It is a software implementation to support threat intelligence, helping an organization generate its own threat intelligence and monitor trends to inform defense mitigation priorities. Integral to the implementation of this measure is the implementation of a configuration database as the primary source of information about the system and its configuration. Mitigation is intended for the customer side and its security and IT department. Mitigation covers not only access in HW but especially to SW platforms including links to the continuity and recovery lanes.	N/A	N/A	N/A	A.12.6	§10, §13, §28
M_50	Update Software [112]	It introduces a requirement for regular software updates to reduce the risk of software misuse, including the development of an update schedule and the introduction of a system for testing and documenting updates. Software updates may need to be scheduled during operational downtime. Mitigation is intended for the customer side and its security and IT department. Mitigation mainly covers protection to SW platforms and data.	N/A	N/A	N/A	A.12.1	§10
M_51	User Account Management [113]	It is the implementation of a central system for documented management, creation, modification, use and permission control of user accounts. Mitigation is the responsibility of the supplier and the configuration engineer. The design must come from the system architect. Mitigation covers access to HW, especially SW platforms.	AC-2	SR 1.3	CR 1.3	A.9.2	§12, §19
M_52	User Training [114]	Continuously educate users on cyber security, to increase user information literacy in order to prevent potential cyberattacks and manipulation by an attacker, in order to reduce the risk of successful spear phishing, social engineering and other techniques that involve user interaction. Mitigation intended for the customer side and its security department. It concerns both HW and SW usage.	AT-2	N/A	N/A	A.6.1	§11, §13

## Data Availability

Not applicable.

## References

[1] Gunduz M.Z., Das R. (2020). Cyber-security on Smart Grid: Threats and Potential Solutions. Comput. Netw..

[2] Pavon W., Inga E., Simani S., Nonato M. (2021). A Review on Optimal Control for the Smart Grid Electrical Substation Enhancing Transition Stability. Energies.

[3] Abrahamsen F.E., Ai Y., Cheffena M. (2021). Communication Technologies for Smart Grid: A Comprehensive Survey. Sensors..

[4] Adamiak M., Falk H., Bishop P., Nair N.K.C. (2022). Wide Area Implementations of IEC 61850 Substation Systems. IEC 61850 Principles and Applications to Electric Power Systems.

[5] Chehri A., Fofana I., Yang X. (2021). Security Risk Modeling in Smart Grid Critical Infrastructures in the Era of Big Data and Artificial Intelligence. Sustainability.

[6] Lázaro J., Astarloa A., Rodríguez M., Bidarte U., Jiménez J. (2021). Survey on Vulnerabilities and Countermeasures in the Communications of the Smart Grid. Electronics.

[7] Zhang H., Liu B., Wu H. (2021). Smart Grid Cyber-Physical Attack and Defense: A Review. IEEE Access.

[8] Pandey J.C., Kalra M., Raj J.S., Kamel K., Lafata P. (2022). A Review of Security Concerns in Smart Grid. Innovative Data Communication Technologies and Application.

[9] Mavale S., Katade J., Dunbray N., Nimje S., Bindhu V., Tavares J.M.R.S., Du K.L. (2022). Review of Cyber-Attacks on Smart Grid System. Proceedings of Third International Conference on Communication, Computing and Electronics Systems.

[10] Krause T., Ernst R., Klaer B., Hacker I., Henze M. (2021). Cybersecurity in power grids: Challenges and opportunities. Sensors.

[11] Mokhor V., Honchar S., Onyskova A. Cybersecurity Risk Assessment of Information Systems of Critical Infrastructure Objects. Proceedings of the 2020 IEEE International Conference on Problems of Infocommunications. Science and Technology (PIC S&T).

[12] Daria G., Massel A. Intelligent System for Risk Identification of Cybersecurity Violations in Energy Facility. Proceedings of the 2018 3rd Russian-Pacific Conference on Computer Technology and Applications (RPC).

[13] Xiao S., Ye Y., Kanwal N., Newe T., Lee B. (2022). SoK: Context and Risk Aware Access Control for Zero Trust Systems. Secur. Commun. Netw..

[14] Mrabet Z.E., Kaaboucha N., Ghazi H.E., Ghazi H.E. (2018). Cyber-security in smart grid: Survey and challenges. Comput. Electr. Eng..

[15] Rawat D.B., Bajracharya C. (2015). Cyber security for smart grid systems: Status, challenges and perspectives. SoutheastCon.

[16] Khan Z.A., Herrmann P. (2019). Recent Advancements in Intrusion Detection Systems for the Internet of Things. Secur. Commun. Netw..

[17] Gunduz M.Z., Das R. A comparison of cyber-security oriented testbeds for IoT-based smart grids. Proceedings of the 2018 6th International Symposium on Digital Forensic and Security (ISDFS).

[18] NIST (2012). NIST Framework and Roadmap for Smart Grid Interoperability Standards Release 2.0.

[19] López G., Moura P., Moreno J.I., Camacho J.M. (2014). Multi-Faceted Assessment of a Wireless Communications Infra-structure for the Green Neighborhoods of the Smart Grid. Energies.

[20] Baul A., Sarker G.C., Sadhu P.K., Yanambaka V.P., Abdelgawad A. (2023). XTM: A Novel Transformer and LSTM-Based Model for Detection and Localization of Formally Verified FDI Attack in Smart Grid. Electronics.

[21] Haq E.U., Xu H., Pan L., Khattak M.I. Smart Grid Security: Threats and Solutions. Proceedings of the 2017 13th Inter-national Conference on Semantics, Knowledge and Grids (SKG).

[22] EU (2016). Directive (EU) 2016/1148 of the European Parliament and of the Council of 6 July 2016 Concerning Measures for a High Common Level of Security of Network and Information Systems across the Union.

[23] Leszczyna R. (2019). Cybersecurity in the Electricity Sector—Managing Critical Infrastructure.

[24] EU (2020). Proposal for a Directive of the European Parliament and of the Council on Measures for a High Common Level of Cybersecurity across the Union, Repealing Directive (EU) 2016/1148.

[25] Hernandez-Ramos J.L., Geneiatakis D., Kounelis I., Steri G., Fovino I.N. (2020). Toward a Data-Driven Society: A Technological Perspective on the Development of Cybersecurity and Data-Protection Policies. IEEE Secur. Priv..

[26] Krzykowski M. (2021). Legal Aspects of Cybersecurity in the Energy Sector-Current State and Latest Proposals of Legislative Changes by the EU. Energies.

[27] Curtis P.D., Mehravari N. Evaluating and Improving Cybersecurity Capabilities of the Energy Critical Infrastructure. Proceedings of the 2015 IEEE International Symposium on Technologies for Homeland Security (HST).

[28] Nazir H.M.J., Han W. (2022). Proliferation of Cyber Situational Awareness: Today’s Truly Pervasive Drive of Cybersecurity. Secur. Commun. Netw..

[29] Sarker E., Halder P., Seyedmahmoudian M., Jamei E., Horan B., Mekhilef S., Stojcevski A. (2021). Progress on the Demand Side Management in Smart Grid and Optimization Approaches. Int. J. Energy Res..

[30] Lyulyov O., Vakulenko I., Pimonenko T., Kwilinski A., Dzwigol H., Dzwigol-Barosz M. (2021). Comprehensive assessment of smart grids: Is there a universal approach?. Energies.

[31] Omitaomu O.A., Niu H. (2021). Artificial Intelligence Techniques in Smart Grid: A Survey. Smart Cities.

[32] Guru D., Perumal S., Varadarajan V. (2021). Approaches towards Blockchain Innovation: A Survey and Future Directions. Electronics.

[33] Alrowais F., Marzouk R., Nour M.K., Mohsen H., Hilal A.M., Yaseen I., Alsaid M.I., Mohammed G.P. (2022). Intelligent Intrusion Detection Using Arithmetic Optimization Enabled Density Based Clustering with Deep Learning. Electronics.

[34] Figueiredo J., Serrão C., de Almeida A.M. (2023). Deep Learning Model Transposition for Network Intrusion Detection Systems. Electronics.

[35] Rabie O.B.J., Balachandran P.K., Khojah M., Selvarajan S. (2022). A Proficient ZESO-DRKFC Model for Smart Grid SCADA Security. Electronics.

[36] Mazhar T., Irfan H.M., Haq I., Ullah I., Ashraf M., Shloul T.A., Ghadi Y.Y., Imran, Elkamchouchi D.H. (2023). Analysis of Challenges and Solutions of IoT in Smart Grids Using AI and Machine Learning Techniques: A Review. Electronics.

[37] Urrea C., Morales C. (2019). Enhancing Modbus-RTU Communications for Smart Metering in Building Energy Management Systems. Secur. Commun. Netw..

[38] Xiao L. (2021). Construction Technology and Quality Control of Power and Electrical Engineering Based on Convolutional Neural Network. Secur. Commun. Netw..

[39] Alazab M., Tang M. (2019). Deep Learning Applications for Cyber Security.

[40] Nguyen T.T., Reddi V.J. (2021). Deep Reinforcement Learning for Cyber Security. IEEE Trans. Neural. Netw. Learn Syst..

[41] Liu H., Lang B. (2019). Machine Learning and Deep Learning Methods for Intrusion Detection Systems: A Survey. Appl. Sci..

[42] Susilo B., Sari R.F. (2020). Intrusion Detection in IoT Networks Using Deep Learning Algorithm. Information.

[43] Thapa N., Liu Z., KC D.B., Gokaraju B., Roy K. (2020). Comparison of Machine Learning and Deep Learning Models for Network Intrusion Detection Systems. Future Internet.

[44] Gupta C., Johri I., Srinivasan K., Hu Y.-C., Qaisar S.M., Huang K.-Y. (2022). A Systematic Review on Machine Learning and Deep Learning Models for Electronic Information Security in Mobile Networks. Sensors.

[45] Alkahtani H., Aldhyani T.H.H. (2022). Developing Cybersecurity Systems Based on Machine Learning and Deep Learning Algorithms for Protecting Food Security Systems: Industrial Control Systems. Electronics.

[46] Akhtar M.S., Feng T. (2022). Detection of Malware by Deep Learning as CNN-LSTM Machine Learning Techniques in Real Time. Symmetry.

[47] Xu C., Liao Z., Li C., Zhou X., Xie R. (2022). Review on Interpretable Machine Learning in Smart Grid. Energies.

[48] Moti M.M.M.A., Uddin R.S., Hai M.A., Saleh T.B., Alam M.G.R., Hassan M.M., Hassan M.R. (2022). Blockchain Based Smart-Grid Stackelberg Model for Electricity Trading and Price Forecasting Using Reinforcement Learning. Appl. Sci..

[49] Piotrowski P., Baczyński D., Kopyt M., Gulczyński T. (2022). Advanced Ensemble Methods Using Machine Learning and Deep Learning for One-Day-Ahead Forecasts of Electric Energy Production in Wind Farms. Energies.

[50] Alrasheedi A., Almalaq A. (2022). Hybrid Deep Learning Applied on Saudi Smart Grids for Short-Term Load Forecasting. Mathematics.

[51] Habbak H., Mahmoud M., Metwally K., Fouda M.M., Ibrahem M.I. (2023). Load Forecasting Techniques and Their Ap-plications in Smart Grids. Energies.

[52] Ibrahim B., Rabelo L., Gutierrez-Franco E., Clavijo-Buritica N. (2022). Machine Learning for Short-Term Load Forecasting in Smart Grids. Energies.

[53] Mazhar T., Irfan H.M., Khan S., Haq I., Ullah I., Iqbal M., Hamam H. (2023). Analysis of Cyber Security Attacks and Its Solutions for the Smart grid Using Machine Learning and Blockchain Methods. Future Internet.

[54] Strom B.E., Applebaum A., Miller D.P., Nickels K.C., Pennington A.G., Thomas C.B. (2020). MITRE ATT&CK: Design and Philosophy. https://attack.mitre.org/docs/ATTACK_for_ICS_Philosophy_March_2020.pdf.

[55] Ackerman P. (2017). Industrial Cybersecurity: Efficiently Secure Critical Infrastructure Systems.

[56] Few C., Thompson J., Awuson-David K., Al-Hadhrami T. A Case Study in the Use of Attack Graphs for Predicting the Security of Cyber-Physical Systems. Proceedings of the 2021 International Congress of Advanced Technology and Engineering (ICOTEN).

[57] Jha A.V., Ghazali A.N., Appasani B., Mohanta D.K., Awad A.I. (2021). Risk Identification and Risk Assessment of Communication Networks in Smart Grid Cyber-Physical Systems. Security in Cyber-Physical Systems, Proceedings of the 2021 International Conference on Advanced Informatics for Computing Research (ICAICR), Gurugram, India, 18–19 December 2021.

[58] The MITRE Enterprise Matrix. https://attack.mitre.org/matrices/enterprise/.

[59] (2019). Information Technology—Security Techniques—Information Security Risk Management.

[60] Common Vulnerability Scoring System Version 3.1 Calculator. Forum of Incident Response and Security Teams, 2015–2022. https://www.first.org/cvss/calculator/3.1.

[61] (2019). Industrial Communication Networks—Network and System Security—Part 3-3: System Security Re-Quirements and Security Levels.

[62] (2019). Security for Industrial Automation and Control Systems—Part 4-2: Technical Security Requirements for IACS Components.

[63] (2014). Information Technology—Security Techniques—Information Security Management Systems—Requirements.

[64] The MITRE Corporation (2022). Access Management. https://attack.mitre.org/mitigations/M0801/.

[65] The MITRE Corporation (2022). Account Use Policies. https://attack.mitre.org/mitigations/M0936/.

[66] The MITRE Corporation (2022). Antivirus/Antimalware. https://attack.mitre.org/mitigations/M0949//.

[67] The MITRE Corporation (2022). Authorization Enforcement. https://attack.mitre.org/mitigations/M0800/.

[68] The MITRE Corporation (2021). Code Signing. https://attack.mitre.org/mitigations/M0945/.

[69] The MITRE Corporation (2022). Data Backup. https://attack.mitre.org/mitigations/M0953/.

[70] The MITRE Corporation (2022). Disable or Remove Feature or Program. https://attack.mitre.org/mitigations/M0942/.

[71] The MITRE Corporation (2022). Execution Prevention. https://attack.mitre.org/mitigations/M0938/.

[72] The MITRE Corporation (2022). Exploit Protection. https://attack.mitre.org/mitigations/M0950/.

[73] The MITRE Corporation (2022). Limit Hardware Installation. https://attack.mitre.org/mitigations/M0934/.

[74] The MITRE Corporation (2022). Mechanical Protection Layers. https://attack.mitre.org/mitigations/M0805/.

[75] The MITRE Corporation (2022). Network Allowlists. https://attack.mitre.org/mitigations/M0807/.

[76] The MITRE Corporation (2022). Network Segmentation. https://attack.mitre.org/mitigations/M0930/.

[77] The MITRE Corporation (2022). Operating System Configuration. https://attack.mitre.org/mitigations/M0928/.

[78] The MITRE Corporation (2022). Out-of-Band Communications Channel. https://attack.mitre.org/mitigations/M0810/.

[79] The MITRE Corporation (2022). Privileged Account Management. https://attack.mitre.org/mitigations/M0926/.

[80] The MITRE Corporation (2022). Restrict File and Directory Permissions. https://attack.mitre.org/mitigations/M0922/.

[81] The MITRE Corporation (2022). Restrict Registry Permissions. https://attack.mitre.org/mitigations/M0924/.

[82] The MITRE Corporation (2022). Restrict Web-Based Content. https://attack.mitre.org/mitigations/M0921/.

[83] The MITRE Corporation (2022). Vulnerability Scanning. https://attack.mitre.org/mitigations/M0916/.

[84] The MITRE Corporation (2022). Watchdog Timers. https://attack.mitre.org/mitigations/M0815/.

[85] The MITRE Corporation (2022). Active Directory Configuration. https://attack.mitre.org/mitigations/M0915/.

[86] The MITRE Corporation (2022). Application Developer Guidance. https://attack.mitre.org/mitigations/M0913/.

[87] The MITRE Corporation (2022). Application Isolation and Sandboxing. https://attack.mitre.org/mitigations/M0948/.

[88] The MITRE Corporation (2022). Audit. https://attack.mitre.org/mitigations/M0947/.

[89] The MITRE Corporation (2022). Boot Integrity. https://attack.mitre.org/mitigations/M0946/.

[90] The MITRE Corporation (2022). Communication Authenticity. https://attack.mitre.org/mitigations/M0802/.

[91] The MITRE Corporation (2022). Data Loss Prevention. https://attack.mitre.org/mitigations/M0803/.

[92] The MITRE Corporation (2022). Encrypt Network Traffic. https://attack.mitre.org/mitigations/M0808/.

[93] The MITRE Corporation (2022). Encrypt Sensitive Information. https://attack.mitre.org/mitigations/M0941/.

[94] The MITRE Corporation (2022). Filter Network Traffic. https://attack.mitre.org/mitigations/M0937/.

[95] The MITRE Corporation (2022). Human User Authentication. https://attack.mitre.org/mitigations/M0804/.

[96] The MITRE Corporation (2022). Limit Access to Resource over Network. https://attack.mitre.org/mitigations/M0935/.

[97] The MITRE Corporation (2022). Minimize Wireless Signal Propagation. https://attack.mitre.org/mitigations/M0806/.

[98] The MITRE Corporation (2022). Mitigation Limited or Not Effective. https://attack.mitre.org/mitigations/M0816/.

[99] The MITRE Corporation (2022). Multi-Factor Authentication. https://attack.mitre.org/mitigations/M0932/.

[100] The MITRE Corporation (2022). Network Intrusion Prevention. https://attack.mitre.org/mitigations/M0931/.

[101] The MITRE Corporation (2022). Operational Information Confidentiality. https://attack.mitre.org/mitigations/M0809/.

[102] The MITRE Corporation (2022). Password Policies. https://attack.mitre.org/mitigations/M0927/.

[103] The MITRE Corporation (2022). Redundancy of Service. https://attack.mitre.org/mitigations/M0811/.

[104] The MITRE Corporation (2022). Restrict Library Loading. https://attack.mitre.org/mitigations/M0944/.

[105] The MITRE Corporation (2022). Safety Instrumented Systems. https://attack.mitre.org/mitigations/M0812/.

[106] The MITRE Corporation (2022). Software Configuration. https://attack.mitre.org/mitigations/M0954/.

[107] The MITRE Corporation (2022). Software Process and Device Authentication. https://attack.mitre.org/mitigations/M0813/.

[108] The MITRE Corporation (2022). SSL/TLS Inspection. https://attack.mitre.org/mitigations/M0920/.

[109] The MITRE Corporation (2022). Static Network Configuration. https://attack.mitre.org/mitigations/M0814/.

[110] The MITRE Corporation (2022). Supply Chain Management. https://attack.mitre.org/mitigations/M0817/.

[111] The MITRE Corporation (2022). Threat Intelligence Program. https://attack.mitre.org/mitigations/M0919/.

[112] The MITRE Corporation (2022). Update Software. https://attack.mitre.org/mitigations/M0951/.

[113] The MITRE Corporation (2022). User Account Management. https://attack.mitre.org/mitigations/M0918/.

[114] The MITRE Corporation (2022). User Training. https://attack.mitre.org/mitigations/M0917/.

